# Spontaneous Emergence of Multiple Drug Resistance in Tuberculosis before and during Therapy

**DOI:** 10.1371/journal.pone.0018327

**Published:** 2011-03-30

**Authors:** Caroline Colijn, Ted Cohen, Ayalvadi Ganesh, Megan Murray

**Affiliations:** 1 Department of Engineering Mathematics, University of Bristol, Bristol, United Kingdom; 2 Department of Epidemiology, Harvard School of Public Health, Boston, Massachusetts, United States of America; 3 Division of Global Health Equity, Harvard Medical School, Boston, Massachusetts, United States of America; 4 School of Mathematics, University of Bristol, Bristol, United Kingdom; St. Petersburg Pasteur Institute, Russian Federation

## Abstract

The emergence of drug resistance in *M. tuberculosis* undermines the efficacy of tuberculosis (TB) treatment in individuals and of TB control programs in populations. Multiple drug resistance is often attributed to sequential functional monotherapy, and standard initial treatment regimens have therefore been designed to include simultaneous use of four different antibiotics. Despite the widespread use of combination therapy, highly resistant *M. tb* strains have emerged in many settings. Here we use a stochastic birth-death model to estimate the probability of the emergence of multidrug resistance during the growth of a population of initially drug sensitive TB bacilli within an infected host. We find that the probability of the emergence of resistance to the two principal anti-TB drugs before initiation of therapy ranges from 10^−5^ to 10^−4^; while rare, this is several orders of magnitude higher than previous estimates. This finding suggests that multidrug resistant *M. tb* may not be an entirely “man-made” phenomenon and may help explain how highly drug resistant forms of TB have independently emerged in many settings.

## Introduction

The World Health Organization estimates that there were approximately 440,000 incident multidrug resistant tuberculosis (MDR TB) cases in 2008. MDR TB is defined by resistance to isoniazid (INH) and rifampin (RIF), the two most important antitubercular antibiotics. The term extensively drug resistant tuberculosis (XDR TB) describes MDR strains with additional resistance to at least one agent in each of the two most effective classes of second line drugs: a fluoroquinolone and an injectable second-line drug (capreomycin, kanamycin, or amikacin). Over 50 countries have reported at least one case of XDR TB and several report that more than 10% of MDR cases are also XDR [1], [2]. XDR has been identified in different TB lineages and strains [3]–[5], suggesting that it has emerged independently on multiple occasions.

Drug resistance in TB is selected when individuals with active tuberculosis are treated with drugs. Although an infectious “dose” of *M. tuberculosis* may consist of only a few bacilli that lodge in distal alveoli of the lung, active pulmonary disease is not usually clinically evident until the population of bacilli has reached a size of 10^8^–10^10^ organisms [6], [7]. Fluctuation tests demonstrate that resistance to specific anti-tuberculosis drugs arises spontaneously at a rate of one in 10^6^–10^9^ cell divisions, depending on the drug [8], [9]. Bacilli resistant to a single drug are therefore highly likely to exist in any detectable TB lesion; accordingly, combination therapy is a mainstay of current TB treatment regimens.

The rate of spontaneous occurrence of MDR TB – the appearance of bacilli resistant to both isoniazid and rifampin before therapy is initiated among those infected with drug sensitive bacilli – has not been measured directly. However, since the mutations that confer resistance to isoniazid and rifampin are independent, it has been assumed that the rate of acquisition of this dual resistance is the product of the two independent mutation rates, around 10^−16^ [10], [11]. These calculations have often been interpreted to imply that multidrug resistance is unlikely to arise spontaneously prior to the administration of drug therapy. Instead, the dominant paradigm for how multidrug resistance arises invokes sequential monotherapy leading to the progressive (stepwise) accumulation of resistance mutations as follows. First, during the time a population of TB bacilli grows from a small inoculum to a sufficient bacterial load to trigger symptoms and diagnosis, mutations occur; a rare drug resistant mutant may then be selected for during treatment. Subsequently, as the drug sensitive population of bacteria dwindles, the mutant population grows to high burden allowing the occurrence of mutations to a second drug; ensuing exposure to that drug now selects for doubly resistant bacilli. Since TB drugs are now rarely administered alone, it is also assumed that exposure to a single drug occurs through "functional monotherapy," i.e. treatment that results in exposing *M. tb* bacilli to a single agent even when multiple drugs are administered. Functional monotherapy may occur when patients do not take their prescribed drugs regularly, when they receive poor quality or counterfeit drugs, when drugs are not properly absorbed through the GI tract or when bacteria grow in protected compartments such as lung cavities where antibiotics either do not penetrate or where their activity is limited by pH or some other functional constraint [7], [12], [13]. The idea that sequential functional monotherapy is required for multidrug resistance to emerge has led to the widely held belief that multidrug resistance is a "man-made phenomena" that results from poorly administered therapy.

As others have noted, the calculation of the per replication probability for the appearance of double mutants does not capture the actual risk that multidrug resistance will emerge in a single symptomatic infection prior to the administration of antibiotics [7], [14]. First, resistance to multiple antibiotics arises not only during one replication event in which a single bacilli acquires two independent mutations, but also can occur during the expansion of a bacterial population from an inoculum even in the absence of drug pressure. If mutations to single drugs occur early during the within-host expansion of a population so that mono-resistant bacilli constitute a major portion of the bacterial population at the time of detection, the likelihood of mutation resulting in resistance to a second drug during the subsequent growth of the population is increased. The within-host emergence of bacilli resistant to multiple drugs depends then not only on the probability of a resistance mutation per replication and the selection pressure provided by drug exposure, but also on the stochastic process that governs the timing of the appearance of single mutants during the growth of the population to a given size.

Secondly, previous estimates of the probability of spontaneous multidrug resistance have also assumed that the population of *M. tb* bacilli at detection size reflects the total number of replication events the mycobacterial population has experienced. In contrast to bacteria grown in culture, which do not experience natural death, *M. tb* bacilli growing in an immunocompetent host are frequently killed through adaptive or innate immune responses and thus a host bacillary population of a given size is likely to have undergone far more replication events than an in vitro population of the same size.

Lastly, the potential fitness costs of resistance may also affect the probability that resistance will be detected in a bacterial population of a given size. Bacteria harboring resistance mutations have been reported to grow more or less quickly than wild type organisms [9], [15]. Since the targets of anti-TB agents include proteins and ribosomal components responsible for transcription, translation and cell wall integrity, resistance mutations may be expected to impede growth or increase the likelihood of cell death in the context of an in vivo infection [16]. In the case of a fitness cost of resistance, the sequential acquisition of multiple deleterious resistance mutations should result in a cumulative fitness deficit that may reduce the likelihood that a strain will evolve extensive drug resistance.

Given these complexities, we developed a mathematical model to estimate the probability that multidrug resistance would emerge spontaneously during the growth of a population of *M. tb* from an initial inoculum to a symptomatic TB infection, given known mutations rates. Using a stochastic birth-death model of the within-host emergence of drug resistant *M. tb*, we show that the probability of emergence of MDR TB is much higher than previously expected, even when combination chemotherapy is reliably delivered.

## Results

### 1) Emergence of monoresistance before treatment

**Figure 1:**
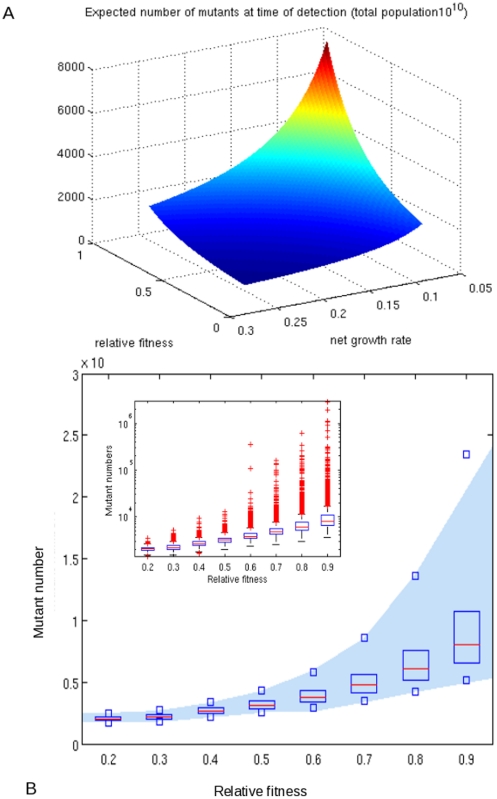
Distribution of INH-resistant mutants at the time of clinical detection of a TB patient. **A** Expected number of INH monoresistant mutants at the time of detection, as a function of relative fitness and net growth rate. **B** The distribution of mutant numbers by relative fitness. Horizontal red lines show the mean values in simulation, and the blue boxes illustrate the inter-quartile range. Small blue squares are the 5^th^ and 95^th^ quantiles. The shaded blue region illustrates the 5^th^–95^th^ quantile for the α-stable distribution.

We first model the emergence of mono-resistance to isoniazid in an immunocompetent host infected by a single drug sensitive *M. tb* bacillus. We assume that in this host, drug sensitive *M. tb* bacilli replicate at a rate λ, die at rate µ and acquire INH resistance-conferring mutations with probability β for each cell division. Prior to diagnosis, the population of sensitive cells grows at net rate λ-µ while the population of resistant cells grows at rate λ_1_−µ_1_ = (λ−µ) ϕ, where ϕ reflects the relative fitness of the resistant mutant. This putative fitness cost can be incurred through reduced growth λ_1_<λ, increased death µ_1_>µ, or both. We assume the bacillary population grows until it reaches size N_f_, the size at which it is likely to produce symptoms and come to clinical diagnosis. The expected total number of resistant bacilli at size N_f_ is the sum of the descendants of all INH resistant mutants arising over the course of the clonal expansion of the bacterial population. (See Supplement S1): 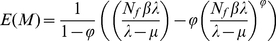
(1)
Figure 1A shows that for a set of parameters specific to the within-host growth of *M. tb* (see Supplement S1), the number of resistant bacilli at the time of detection varies with both the relative fitness of the resistant mutants and with the net growth rate. The number of mutants is higher when the net growth is lower because more cell turnover has occurred at a given detection size, thereby creating more replication events during which mutations could occur. In contrast to previous estimates that there will be hundreds of INH resistant bacilli present by the time an infection becomes symptomatic (i.e. reaches size 10^10^) [7], [17], [18], [19], we estimate mean numbers of INH resistant bacilli an order of magnitude higher.

Our results also demonstrate that the number of resistant bacteria at the time of clinical detection is extremely variable (Figure 1B). For example, in the biologically realistic scenario that the fitness cost is less than 1/2, i.e. that ϕ>½, we find that the distribution of mutant numbers has power-law tails with exponent 1/ϕ (see Supplement S1). This highly skewed distribution means that some individuals have many times the average number (5000) of INH-resistant mutants as illustrated in Figure 1.

### 2) Emergence of multidrug resistance before treatment

**Figure 2:**
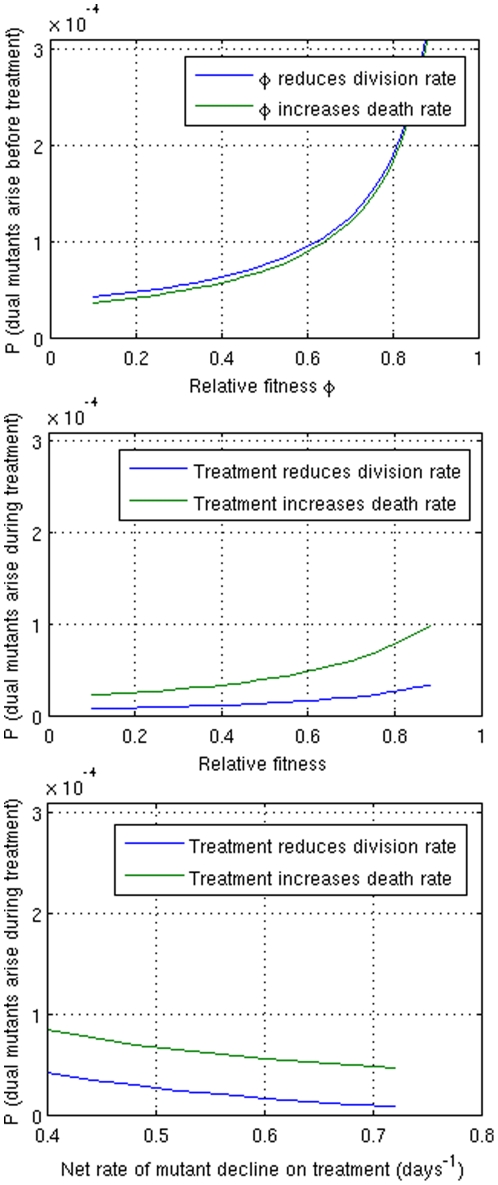
Probability of dual resistance (p_dual_). Numbers are based on β = 2.25×10^−8^ (isoniazid resistance) and β_12_ = 3.3×10^−9^ (rifampin resistance) [7], [8] **A** Before treatment begins, showing that when mutants have higher relative fitness there is more mutant growth corresponding to a higher probability that dual resistance will arise; **B** After initiation of therapy; **C** After initiation of therapy, showing the dependence on the net rate of decline. During the decline of the bacterial population during therapy there may be some turnover, although the death rate will be greater than the division rate. In particular, if treatment increases the death rate but does not affect the division rate there may be substantial turnover, resulting in a larger probability that dual resistance may arise (green vs blue lines in panels B and C). A more rapid net decline results in fewer births and a lower risk of resistance. In each panel we allow the appearance of resistance to isoniazid or rifampin to occur first, but the rapid bactericidal effect of INH means that the net decline of INH-sensitive bacteria is much more rapid than that of INH-resistant ones, and isoniazid resistance is thus more likely to be observed before rifampin resistance in treated patients. [19]

Using this model, we estimated the probability that multidrug resistant cells arise prior to TB detection based on the expected number of divisions of singly-resistant cells prior to the population reaching size N_f_. The probability that multiple resistance ever emerges during the modeled growth of the singly-resistant population is given by: 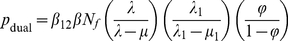
(2)This suggests that given known mutation rates, the probability of a spontaneously occurring MDR TB bacillus p_dual_ arising during clonal expansion ranges from 1 in 3000 to 1 in 20,000, and increases with relative fitness (Figure 2A). If we assume that some of these bacilli will die prior to replication, the risk of dual resistance at the time of detection is lower by a factor of approximately ten (based on an extinction probability of µ_1_/λ_1_).

Since recent evidence suggests that some isoniazid and rifampin resistance mutations (such as the S315T mutation in *katG*) bear little if any fitness cost [20], [21], [22], [23], the probability that a second resistance-conferring mutation will occur during the modeled growth of an isoniazid resistant strain with this mutation may be as high as 0.0005, or 1 in 2000. These numbers are in stark contrast to previous estimates of the risk of dual resistance of approximately 1 in 10,000,000 cases before the introduction of therapy [7], [17], [18], [19].

### 3. Emergence of multidrug resistance during treatment

Dual resistance may also arise during combination therapy while singly-resistant cells are killed through treatment. Although four drugs are typically included in the first two months of tuberculosis treatment, i.e. the initiation phase, only two drugs are used during the subsequent continuation phase; consequently, continuation therapy may efficiently select for dual resistance in bacilli already resistant to one agent. Most active TB agents including isoniazid, rifampin, streptomycin and fluoroquinolones are bactericidal and for these, we assume that treatment increases the death rate. Others such as ethambutol and PAS are bacteriostatic and work through reducing the rate of bacillary division. Although we assume that combination therapy will kill both drug sensitive cells and singly-resistant mutants, continued growth of at least some resistant mutants may occur during this decline. This gives an estimated probability of dual-resistance arising as: 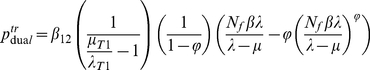
(3)(See the Supplement S1 for a derivation).

Figure 2 shows that dual resistance may arise even during completely effective treatment in as many as 1 in 10,000 cases (p = 3×10^−4^) and could emerge either before or during treatment in as many as 1 in 2500 cases (based on adding the probabilities in Figure 2A and 2B). This is consistent with the fact that combination therapy has been shown to be highly effective [7], but in settings in which TB incidence is high, spontaneously-emerging multiple resistance may account for occasional treatment failures.

The risk of emergence of multiple drug resistance is higher when treatment increases the death rate than it is when treatment reduces the division rate, even when the net rate of decline is the same. This is because reducing the division rate reduces the turnover of bacilli and thus the number of mutations. Furthermore, the probability of dual resistance emerging during treatment is far greater when treatment is less rapidly bactericidal, reflecting the fact that more turnover may happen before the population of single mutants is eliminated. This is consistent with the observation that rifampin resistance is far more likely to emerge from a population of isoniazid-resistant mutants than the other way around [17], since the rapid bactericidal action of isoniazid should suppress mutation among rifampin resistant mutants but would not affect pre-existing isoniazid-resistant mutants.

## Discussion

Using a stochastic birth-death model of the within-host emergence of drug resistance, we find that the probability that resistance will develop during the clonal expansion of an initially sensitive TB infection is orders of magnitude higher than previously estimated. This result suggests that even in settings where appropriate treatment is available and properly administered, MDR tuberculosis can emerge during the within host growth of the bacterial population. Since we model the idealized scenario in which MDR bacilli arise from a completely sensitive original infection, our results represent a lower bound on the frequency of MDR; in a real world setting lapses in effective chemotherapy and the transmission of resistant strains are also major contributors to the burden of drug resistant TB. Although we have here modeled the emergence of isoniazid mono-resistant and MDR strains, TB mutates to develop resistance to other anti-tuberculosis agents at rates that are similar or even higher than to isoniazid [7], [8] and therefore we expect that resistance to these drugs also exists at high numbers in any advanced TB infection. Furthermore, if mono-resistant strains are transmitted to a new host and lead to secondary infections, our results predict that a range of different dually resistant bacilli would arise, and eventually be found in large numbers. Similarly, combination therapy in patients with MDR would be expected to result in the selection of dually resistant bacilli at high risk of developing further resistance.

These results differ from previous expectations because we allowed dual resistance to emerge during the expansion of a population of TB bacilli in an immunocompetent host. This formulation of the problem allowed us to consider the scenario in which a second drug resistance mutation occurs during the growth of a population of singly resistant mutants rather than assuming that dual mutations occur during a single replication event. Zheng has previously noted that classic estimates of the probability of the occurrence of double mutants ignore the fact that sequential random mutations are expected in a growing population of bacteria[14].

We also assumed that in contrast to bacterial growth in vitro, some pathogenic bacteria in an immunocompetent host will be killed through an immune response and, thus, the number of replication events required for a population to reach a given size is greater than if death had not occurred [24]. Accordingly, since the total number of replication events is lower in vitro, the risk of dual resistance observed among bacteria grown in the laboratory may not reflect the risks of dual resistance emerging in hosts.

High frequencies of mutation have been observed in isolates from chronic clinical infection with other bacterial pathogens including *Stenotrophomonas maltophilia* [25] and *Pseudomonas aeruginosa* [26]. Furthermore, there is evidence that sub-lethal concentrations of antibiotics can induce mutagenesis [27]. Our model does not include this mechanism to account for the emergence of resistance, largely because the evidence supporting hypermutability in *M. tuberculosis* is mixed [28], [29], [30]. However, if *M. tb* bacilli do increase their rate of mutation either during growth of the initial infection or following exposure to antibiotics, we would expect the emergence of multidrug resistance during treatment to be accelerated and the overall frequency of the emergence of MDR to be even higher than we have estimated here.

Our results may also underestimate the frequency of spontaneous resistance because we assume a fitness cost of the drug resistant strain. Recent evidence suggests that the initial fitness costs of some drug resistance mutations can be rapidly compensated by secondary mutations that restore normal function [31], [32], [33]. In this case, fitness costs may be transient and the probability of the emergence of resistance will thus be at the upper end of the spectrum presented here. Interestingly, the acquisition of a fitness-restoring mutation in a singly resistant mutant during clonal expansion of the TB population should follow very similar dynamics to those we have presented here for dual resistance. On the other hand, if only a fraction of mutations observed during in vitro growth of *M. tuberculosis* are actually found in clinical strains [34], the mutation rates cited in this study are higher than those that would reflect the acquisition of clinical resistance. In this case, both the standard approach and our model would predict correspondingly lower risk of emergence of multiple resistance, but our estimates would still be orders of magnitude higher.

In summary, using a simple model of an initially drug sensitive *M. tb* infection, we estimated that the probability that MDR exists at the time of diagnosis may be 1000–10,000 times higher than previously suggested. Based on these results, we anticipate that dually resistant strains will be present in a small minority of patients even prior to treatment, and, for this subset of patients, standard drug regimens administered properly will likely result in the selection of pre-existing dual resistance. Our results point to a mechanism, distinct from functional monotherapy, which can explain how highly drug resistant *M. tb* may emerge in the context of combination therapy. We propose that this may account for the repeated independent emergence of MDR and by extension, XDR tuberculosis, both in individuals and across a wide range of geographical settings.

## Methods

We model infection arising as a result of infection with one drug sensitive bacillus. This bacillus divides and at each division may create a resistant mutant with probability β, which for isoniazid resistance is on the order of 10^−8^ [8]. In a clinical TB infection there may be up to 10^10^ bacilli [9], so we expect that several mutation events will have occurred. We wish to find the distribution of the total number of mutants, including descendants of early mutation events. This problem is closely related to the Luria-Delbruck theory [35], [36], [37] and subsequent mathematical models [38], [39], [40], [41], [42]; however, these typically either do not include cell death or assume that resistant mutants divide and die at the same rates as sensitive cells [22], [24], [36]. Furthermore, we develop an intuitive approach to the distributional estimate, avoiding the use of generating functions. This approach is particularly useful in the description of within host TB dynamics, as TB infections are large enough that the population of single mutants is expected to be substantial and therefore a focus on the probability of single resistance is less relevant than in related work on the emergence of resistance in cancer [43]. We assume that there is a fitness cost associated with drug resistance, i.e., that the relative fitness of the mutant compared to the sensitive strain is smaller than 1 (though it may be close to 1 if the fitness cost is low). Combined with the rarity of mutations, this ensures that mutants will comprise only a very small proportion of the population, so that the time when sensitive cells reach the detection size is very close to the time that the entire population reaches that size[44] Also, drug sensitivity testing would be very unlikely to detect resistant mutants under these assumptions.

### Mean mutant numbers

We compute the mean single mutant numbers as a function of the relative fitness and growth and death rates of the sensitive cells by finding the mean growth of the sensitive cell population between any two mutation events. We then relate the net growth of mutant cells to the net growth of sensitive cells via the relative fitness (see Supplement S1); the mean mutant population at the time of detection is the sum of the descendants of all of the mutants that arose during the growth of the sensitive population.

### Distribution of mutant numbers

We find the distributional estimate for the total mutant number at the time of detection essentially by showing that mutations occur uniformly over the growth of the sensitive population. We then show that the number of descendants of an individual mutant is a random variable *Q* which has a Pareto distribution, whose complementary cumulative distribution behaves like x^1/ϕ^; this gives rise to the infinite-variance distributions for ϕ>½. The total mutant population is composed of a sum of *J* i.i.d quantities distributed as *Q*, and *J* itself is Poisson. This, together with the fact that the sensitive cell population at the time of detection is large enough that many different mutants have arisen, means that the mutant numbers at the time of detection are approximated by an α-stable distribution. See the Supplement S1 for the details of this derivation.

### Dual mutants

Both before and after treatment begins, we compute the probability that a dually resistant mutant arises by finding the expected number of arrivals or divisions of singly resistant mutant cells. Each time a singly resistant cell is created there is a small probability that it is dually resistant, and this combined with the results on mean mutant numbers yields the expression above for the probability of dual resistance. Further details are presented in the Supplement S1.

## Supporting Information

Supplement S1 (PDF)
