# Supplementary material for: Spontaneous Emergence of Multiple Drug Resistance in Tuberculosis before and during Therapy
Source: PLoS One. 2011 Mar 30;6(3):e18327. doi: 10.1371/journal.pone.0018327 (PMC3068161; doi:10.1371/journal.pone.0018327)
Supplement: Supplement S1 — (PDF) [file pone.0018327.s001.pdf]

Supplement S1

We model infection arising as a result of one drug-sensitive bacillus which divides and at each division may create a resistant mutant with probability β, which for isoniazid resistance is on the order of 10^{-8} [1]. In a clinical TB infection there may be up to 10^{10} bacilli [2], so we expect that several mutation events will have occurred. We wish to find the distribution of the total number of mutants, including descendants of early mutation events. This problem is closely related to the Luria-Delbruck theory [3, 4, 5] and subsequent mathematical models,including [6, 7, 8, 9, 10]; however, these typically either do not include cell death or assume that mutants divide and die at the same rates as the sensitive cells [4, 5, 11]. Furthermore we develop an intuitive approach to the distributional estimate, avoiding the use of generating functions. This approach is particularly useful in the TB setting, as TB infections are large enough that the population of single mutants is expected to be considerable so that a focus on the probability of single resistance is less appropriate than in related work on the emergence of resistance in cancer [12]. We assume that there is a fitness cost associated with drug resistance, i.e., that the relative fitness of the mutant compared to the sensitive strain is smaller than 1 (though it may be close to 1 if the fitness cost is low). Combined with the rarity of mutations, this ensures that mutants will comprise only a very small proportion of the population, so that the time when sensitive cells reach the detection size is very close to the time that the entire population reaches that size [13, 14]. Also, drug sensitivity testing would be very unlikely to detect resistant mutants under these assumptions.

Mean mutant numbers

The net growth of the population of sensitive bacterial cells is given by the difference between their average rate of division, which we denote λ, and their rate of death μ. Similarly, any resistant mutants that arise during growth may die out, or may divide; we denote the rates of division and death of mutants as λ_1 and μ_1 respectively. Resistance mutations typically result in some "fitness cost" which reduces mutants' replicative capacity relative to drug-sensitive bacilli; we therefore define a relative fitness parameter $\phi = (\lambda_1 - \mu_1)/(\lambda - \mu)$ to be the ratio of the net growth rates of mutant bacilli to sensitive ones. We denote the mutation probability per cell division as β.

Let K denote the random number of cell divisions until the first mutant, ignoring for now the possibility of extinction. We have

$$P(K = k) = (1-\beta)^k \beta, \quad k = 1, 2, 3, ... \text{ and } E(K) = \frac{1}{\beta}.$$

The former is just the probability that the first k divisions did not have a mutation event and the $(k+1)^{\text{th}}$ does. Now let X_i denote the number of deaths between cell divisions $i-1$ and i. The probability that the next death occurs before the next division is $\mu/(\mu+\lambda)$. This means that $X_i \sim \text{Geom}(\frac{\lambda}{\lambda+\mu}) - 1$, i.e., $P(X_i = k) = \frac{\lambda}{\lambda+\mu}(\frac{\mu}{\lambda+\mu})^k$ for $k \geq 0$. This is an approximation, in that the X_i have truncated geometric distributions, as the possible number of deaths is limited by the current population size. However, as the population grows large, this will not affect the results that follow. The number of cell deaths before the first mutation is $\sum_{k=1}^{K} X_k$, where the number of terms is random. The population size at the time of the first mutation is then given by

$$N_1 = K - \sum_{k=1}^{K} X_k = K\left(1 - \frac{1}{K}\sum_{k=1}^{K} X_k\right). \tag{1}$$

Since the X_i are identically distributed, and independent of K, we have

$$E(N_1) = E(K)(1 - E(X_1)) = \frac{\lambda - \mu}{\lambda\beta}. \tag{2}$$

With parameters reflecting growth from the initial infection to a high-grade infection in approximately 5 months and a daily cell cycle, $E(N_1) = 6 \times 10^6$. The same reasoning applies to the change in the sensitive population between the times of the kth and and $(k+1)^{\text{th}}$ mutations, for any k. On average, we expect that the sensitive population grows by an additional $E(N_1)$ before the arrival of the next mutant, so that $E(N_k) = kE(N_1)$ is the expected size of the sensitive population at the arrival time of the kth mutant.

Let $R_j(t)$ represent the size, at time t, of the mutant population descended from the jth mutation from the sensitive population (the "j^{th} clone" for short). We set $R_j(t) = 0$ for $t < \tau_j$, the (random) time of the jth mutation. The key feature of our approach is to write the total mutant number as a sum of these independent population sizes.

We have $E(R_j(t)|\tau_j) = e^{(\lambda_1-\mu_1)(t-\tau_j)}$, so that the expected total mutant number $E(M(t))$ is

$$E(M(t)|\tau_1, \tau_2, \ldots) = \sum_{j=0}^{\infty} e^{(\lambda_1-\mu_1)(t-\tau_j)}.$$

Between times τ_1 and τ_k, the sensitive population has grown by a factor of k in expectation. The mutant strain grows ϕ times as fast, so we expect the size of the population descended from the first mutant to have grown by a factor k^ϕ. (More precisely, we need the expectation of the random variable $(N_k/N_1)^\phi$, but are approximating this by $(E(N_k)/E(N_1))^\phi$. The approximation is good for large k. More refined estimates are presented in the next section.) Similarly, between times τ_j and τ_k, the sensitive population has grown by a factor k/j on average, so the mutant arising at τ_j would have grown by a factor $(k/j)^\phi$. At time τ_k, the expected value of the total mutant population is thus

$$E(M(T_k)) = \sum_{j=1}^{k} \left(\frac{k}{j}\right)^\phi = k^\phi \sum_{j=1}^{k} \frac{1}{j^\phi}. \tag{3}$$

We expect J, the maximum value k will reach before the sensitive population hits the detection size, to be large enough that the sum can be approximated by an integral, yielding

$$E(M(T_J)) \leq k^\phi \left(1 + \int_0^{k-1} \frac{1}{(1+x)^\phi} dx\right) = \frac{J - \phi J^\phi}{1-\phi}, \quad \phi \neq 1. \tag{4}$$

As the sensitive population is approaching the detection size, mutations are occurring very frequently, so that the time between the last mutation and the detection time is small, and we ignore it. We expect $J = N_f/E(N_1)$; substituting from Eq. (2) yields Eq. 1 in the main text.

Distribution of mutant numbers

The fact that β is small means that N_1 is very close to a multiple $(\lambda-\mu)/\lambda$ of a Geom(β) random variable. More precisely, if we define scaled variables $Z_k = \frac{\lambda\beta}{\lambda-\mu}(N_k - N_{k-1})$, then the Z_k are independent and identically distributed, and Z_1 converges to an exponentially distributed random variable with parameter 1 (denoted Exp(1) as β tends to zero. To see this, we first note that, for K given by equation (1), βK converges in distribution to an Exp(1) random variable as β tends to zero. This can be seen by direct calcuation:

$$P(\beta K \geq x) = \sum_{n=\lceil x/\beta \rceil} P(K = n) = (1-\beta)^{\lceil x/\beta \rceil} \to e^{-x} \text{ as } \beta \to 0.$$

Next, we observe that K tends to infinity in probability as β tends to zero, and so $\frac{1}{K}\sum_{j=1}^{K} X_j$ tends to $E[X_1] = \mu/\lambda$. It now follows by Slutsky's theorem [15] that $\beta K(1 - \frac{1}{K}\sum_{j=1}^{K} X_j)$ converges in distribution to an Exp$(1-\frac{\mu}{\lambda})$ random variable, and hence that

$$Z_1 = \frac{\lambda\beta}{\lambda-\mu} K\Big(1 - \frac{1}{K}\sum_{j=1}^{K} X_j\Big)$$

converges in distribution to an Exp(1) random variable.

Since the relevant mutation rates are small compared to the net growth of the sensitive population, $E(N_1)$ is large enough that we can approximate the growth of the sensitive cells as deterministic from this point onwards: $N(t) = N_1 e^{(\lambda-\mu)(t-\tau_1)}$. Let τ_k represent the time at which the kth mutation occurred, and $T_k = \tau_{k+1} - \tau_k$. Let $\zeta_k = \sum_{j=1}^{k} Z_j$. Conditional on the sizes N_k, we can use the approximation of deterministic growth of the sensitive population to write

$$T_k \approx \frac{1}{\lambda-\mu} \log \frac{\zeta_{k+1}}{\zeta_k}. \tag{5}$$

The approximation can again be made precise as a limiting result as β tends to zero.

If we define

$$W_j(t) = e^{-(\lambda_1-\mu_1)t} R_j(\tau_j + t)$$

then it can be readily verified that W_j is a non-negative martingale. Hence, it converges to a random variable of unit mean as t approaches infinity. We are interested in the mutant population at a time τ_f when the sensitive population reaches a detection size. This can be expressed as

$$M(\tau_f) = \sum_{j:\tau_j \leq \tau_f} e^{(\lambda_1-\mu_1)(\tau_f-\tau_j)} W_j(\tau_f - \tau_j)$$

Using Eq. (5),

$$\begin{aligned} M(\tau_f) &= \sum_{j:\tau_j \leq \tau_f} \left(\frac{\zeta(\tau_f)}{\zeta_j}\right)^{\phi} W_j(\tau_f - \tau_j) \\ &= \sum_{j:\tau_j \leq \tau_f} \left(\frac{\zeta(\tau_f)}{\zeta_j}\right)^{\phi} W_j\left(\frac{1}{\lambda-\mu} \log \frac{\zeta(\tau_f)}{\zeta_j}\right), \end{aligned} \tag{6}$$

where we write $\zeta(\tau_f)$ to denote $\frac{\lambda\beta}{\lambda-\mu} N(\tau_f)$, and $N(\tau_f)$ is the(possibly random) size of the sensitive population at the detection threshold. Now recall that $Z_1, Z_2 \ldots$ are i.i.d Exp(1) random variables, and $\zeta_j = \sum_{k=1}^{j} Z_j$. Thus, the ζ_j are the points of a unit rate Poisson process when we measure time in terms of the (scaled) size of the sensitive population. Consequently, conditional on $\zeta(\tau_f)$, the number J of mutations up to τ_f is a Poisson random variable with mean $\zeta(\tau_f)$. Moreover, conditional on J, the variables $\frac{\zeta_j}{\zeta(\tau_f)}, \quad j = 1, \ldots, J$ are distributed like the order statistics of J iid random variables distributed uniformly on $[0, 1]$. Intuitively this expresses the fact that mutations are occurring uniformly with respect to the growth of the sensitive population (at a rate of one mutation per growth by $E(N_1)$), so a Poisson-distributed number of them will occur over a fixed net growth. We can then rewrite Eq. (6) as

$$M(\tau_f) = \sum_{j=1}^{J} U_j^{-\phi} W_j \left(\frac{-\log U_j}{\lambda-\mu}\right) \tag{7}$$

where J is a Poisson random variable and U_i are iid random variables, independent of J, uniformly distributed on $[0, 1]$.

The long tails in mutant numbers arise because of the $U_j^{-\phi}$ term in Eq. (3), which has an $x^{1/\phi}$ tail: $P(U_j^{-\phi} > x) = x^{-1/\phi}$. In particular, when $\phi > \frac{1}{2}$, $U_j^{-\phi}$ has infinite variance. Moreover, we can show the following:

Theorem 1 *Let* $Q = U_j^{-\phi} W_j \left(\frac{-\log U_j}{\lambda-\mu}\right)$, *where* W_j *is defined as above. Then,*

$$c := \lim_{x\to\infty} x^{1/\phi} P(Q > x) = \Big(\frac{\lambda_1}{\lambda_1-\mu_1}\Big)^{\frac{1}{\phi}-1} \Gamma\Big(\frac{1}{\phi}+1\Big). \tag{8}$$

The proof is given at the end of the Supplementary Material.

When J is relatively large, the infinite-variance tail behaviour for $\phi > 1/2$ means that the distribution of mutant numbers can be approximated by an appropriately scaled α-stable distribution; this allows the computation of the theoretical quantiles[16] shown in Figure 1B; we have used the program STABLE of J. P. Nolan:[1]

$$q_p \approx \frac{q_p^{0,1} + b_J}{a_J} \tag{9}$$

where $q_p^{\alpha,1,0,1}$ is the p'th quantile of the centred α-stable distribution with exponent $\alpha = 1/\phi$, skew parameter 1, mean 0 and shape parameter 1. We have[16]

$$a_J = \left(\frac{2\Gamma(\alpha)\sin\left(\frac{\pi\alpha}{2}\right)}{\pi c} \right)^{1/\alpha} J^{-1/\alpha}, \quad b_J = n a_n E\left(U_j^{-\phi} W_j \left(\frac{-\log U_j}{\lambda - \mu} \right) \right)$$

and c is determined by the limiting tail behaviour as in Eq. (8). For high values of relative fitness, the exponent α approaches 1, and the convergence to the α-stable distribution in the tails is slow, requiring very large values of J. Using parameters modelling INH resistance, $J \sim 1700$; numerical evidence suggests that this approximation is good for $\phi < 0.85$ but that after that, the upper quantiles given by Eq. (9) are underestimates (see blue squares in Figure 1B). Figure S1 illustrates the difference in the distribution of mutant numbers for $\phi < \frac{1}{2}$ and $\phi > \frac{1}{2}$ in our numerical simulations.

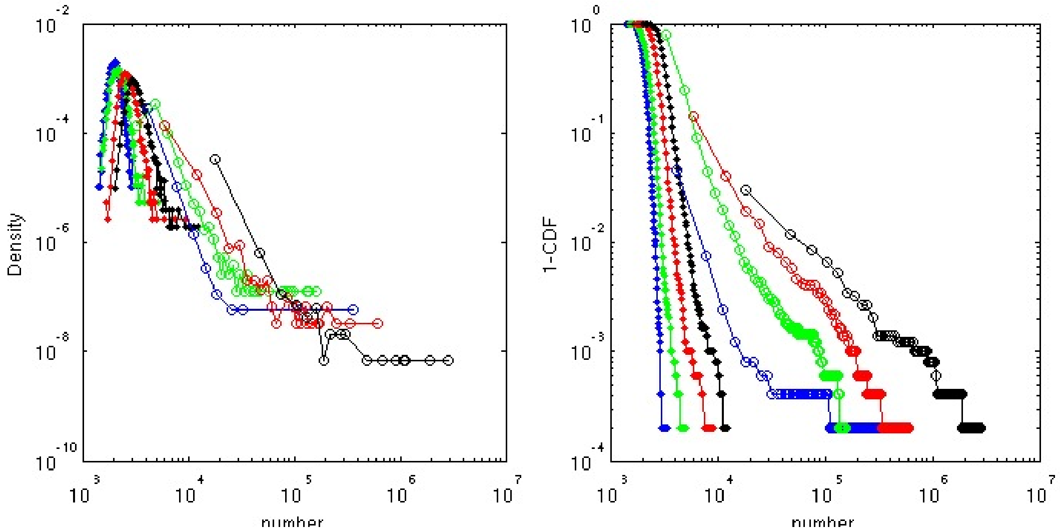

Figure S1: Numerically determined mutant density (left) and 1-cumulative distribution function (right) illustrating the emergence of power-law tails for $\phi > \frac{1}{2}$.

Dual mutants

We estimate the chance of dual resistance arising from the population of singly-resistant mutants during their growth (and, below, during their decline on treatment). Each time a singly-resistant bacillus divides there is a probability (denoted β_{12}) of a second resistance mutation arising; if this happens, the resulting cell could give rise to a population of descendants or it could go extinct. However, while there are significant numbers (thousands) of singly-resistant bacilli at the time of detection, the mutation rates to dual resistance remain low; we therefore estimate simply the probability that dual resistance will arise at all during the growth of the infection.

[1]The program STABLE is available from J. P. Nolan's website: academic2.american.edu/~jpnolan

The expected number of divisions (ie opportunities for dual resistance to arise) of singly-resistant cells in the jth clone is given by

$$B_j = \int_{\tau_j}^{\tau_J} \lambda_1 e^{(\lambda_1-\mu_1)(t-\tau_j)} dt = \frac{\lambda_1}{\lambda_1 - \mu_1}(e^{(\lambda_1-\mu_1)(\tau_J-\tau_j)} - 1)$$

Again conditioning on the sizes N_k of the sensitive population at the time of the kth arrival we find that the total number of divisions of singly-resistant bacilli is

$$B = \sum_{j=1}^{J} B_j = \frac{\lambda_1}{\lambda_1 - \mu_1} \sum_{j=1}^{J} \left(\left(\frac{N_f}{N_j} \right)^{\phi} - 1 \right) \tag{10}$$

and the expected number of births is

$$E(B) = \left(\frac{\lambda_1}{\lambda_1 - \mu_1} \right) E(J) \int_0^1 (U^{-\phi} - 1) du = E(J) \left(\frac{\lambda_1}{\lambda_1 - \mu_1} \right) \left(\frac{\phi}{1 - \phi} \right)$$

where $E(J) = \zeta(\tau_f) = \frac{\lambda\beta}{\lambda-\mu} N(\tau_f)$. The probability that dual resistance arises is

$$p_{\text{dual}} = 1 - (1 - \beta_{12})^{E(B)} \sim e^{-\beta_{12} E(B)} \sim \beta_{12} E(B).$$

This gives Eq. 2 of the main text, and values are illustrated in Figure 2A showing the dependence on the relative fitness ϕ, with division and death rates reflecting daily division and net growth to detection size in 5 months.

So far, this discussion has focused on the probability of dually resistant mutants arising from one kind of singly-resistant mutants only, but either single resistance mutation may occur first; the combined probability of seeing dual resistance before treatment is very close to the sum of the probabilities for each route. These will differ depending on the fitness costs and mutation rates of the two types of single mutants. For INH and rifampin resistance, if the fitness costs are the same, and the mutations are independent, the probability of duals emerging before treatment is the same through either route; Figure 2 shows the sum of the probabilities, thereby allowing dual resistance to emerge through either singly-resistant type first, under the assumption that fitness costs are the same.

Now consider the risk of dual resistance emerging during treatment. In any multidrug regimen, we make the conservative assumption that the sensitive cells essentially cease to divide, and therefore neglect the chance that singly resistant mutants arise and create dual resistance. Rather, we explore the likelihood that dual resistance arises from the populations of single mutants that are already present when treatment begins. Suppose that multiple treatment will eventually kill the entire population of single mutants. During their (stochastic) decline, a death is now more likely than a division, and the average number of deaths is a factor $\frac{\mu_{T1}}{\lambda_{T1}}$ more than the number of divisions. Because we assume that all cells will ultimately die, the population's net decline during treatment is simply M_0 – the number that occurred before treatment began. This gives the number of births as $B = \frac{1}{\mu_{T1}/\lambda_{T1}-1} M_0$ where M_0 is the number of single mutants present when treatment begins; as above, the fact that β_{12} is small means that $p^{tr}_{\text{dual}} \sim \beta_{12} E(B)$, which gives

$$p^{tr}_{\text{dual}} \sim \beta_{12} \frac{1}{\frac{\mu_{T1}}{\lambda_{T1}} - 1} E(M),$$

and substituting for the expected value $E(M)$ of the number of singly resistant mutants at the time of detection yields Eq. 3 of the main text. Figure 2B and C illustrate this result and its dependence on the relative fitness and on the net rate of decline during treatment. Furthermore, the probability of dual resistance emerging during treatment is greater when treatment kills bacteria less rapidly

because this allows for more turnover before the population becomes extinct. For this reason, we would expect to see rifampin resistance emerge from a population of INH-resistant mutants rather than the other way around, because of the rapid bactericidal action of INH. [17].

Results for the long-tailed distribution of mutant numbers have been obtained in several studies on the Luria-Delbruck distribution [12, 4, 18]. Here, our explicit computation of the likelihood of dual resistance, our elucidation of the dependence on mutation rates and the mechanism through which the fitness costs are inferred, and our reliance on the uniformly distributed R.V. U as opposed to generating functions add to this understanding and make clear its application to multiple drug resistance in tuberculosis infections.

Simulations

We also estimate the distribution of mutant numbers by simulating the growth of the bacterial population as follows. Let N be the population of sensitive cells with $N(0) = 1$. While $N(t)$ is below a threshold value (here 100), we use a monte carlo method to decide $N(t+1)$: ie choose $N(t)$ random numbers a_k uniformly distributed on [0, 1]. For each $k = 1, .., N(t)$, the k'th cell grows if $a_k < \lambda_k \Delta t$, dies if $\lambda\Delta t < a_k < \lambda\Delta t + \mu\Delta t$, and so on, dividing the interval up [0,1] appropriately. This method is prohibitive as N grows large, but for large N, the net growth will have a mean of $N(\lambda - \mu)\Delta t$; each cell in the large population can be represented as either growing with probability $\lambda\Delta t$, dying with probability $\mu\Delta t$ or neither. We therefore choose the number of new divisions and deaths to be Poisson-distributed numbers with the corresponding means, and assign $N(t+1) = N(t) + \text{Divisions} - \text{Deaths}$. Similarly, we choose Poisson-distributed numbers of new mutants with the correct means when the mutant numbers exceed the threshold. We continue this process until the expected value of N is 10^{11}. Then we can the time at which the sensitive population actually reached the detection size $N_f = 10^{10}$ in all simulations where the sensitive population did not become extinct, and collect the mutant numbers at those times. The resulting data are shown in Figure 1.

Proof of Theorem 1

Recall that $Q = U_j^{-\phi} W_j(-\frac{\log U_j}{\lambda-\mu})$, U_j is uniform on $[0, 1]$, and $W_j(t) = e^{-(\lambda_1-\mu_1)t} R_j(t+\tau_j)$, where $R_j(t+\tau_j)$ is the number of descendants alive at time $t+\tau_j$ of the mutant that arose at time τ_j. Set $\tau_j = 0$ without loss of generality. Note that if define $T = -\log U_j/(\lambda-\mu)$, then T is an Exp$(\lambda-\mu)$ random variable, and $U_j^{-\phi} = e^{\phi(\lambda-\mu)T} = e^{(\lambda_1-\mu_1)T}$, since $\phi = \frac{\lambda_1-\mu_1}{\lambda-\mu}$ is the fitness penalty. Hence $Q = R_j(T)$. Here, $R_j(T)$ is the population size in a branching process with birth rate λ_1, death rate μ_1 and initiated by a single individual, at a random time T which is exponentially distributed with parameter $\lambda - \mu$. The exact distribution for such a branching process at any fixed time is known, and we shall use it to compute the tail estimate for Q. Specifically, it has been shown[18] that

$$p_n(t) := P(R_j(t) = n) = \frac{(\lambda_1-\mu_1)^2 e^{-(\lambda_1-\mu_1)t}}{[\lambda_1 - \mu_1 e^{-(\lambda_1-\mu_1)t}]^2} \Big(\frac{\lambda_1 - \lambda_1 e^{-(\lambda_1-\mu_1)t}}{\lambda_1 - \mu_1 e^{-(\lambda_1-\mu_1)t}}\Big)^{n-1}. \qquad (11)$$

Hence, for positive integers n, we have

$$P(Q > n | T = t) = \sum_{m=n+1}^{\infty} p_n(t) = \frac{\lambda_1 - \mu_1}{\lambda_1 - \mu_1 e^{-(\lambda_1-\mu_1)t}} \Big(\frac{\lambda_1 - \lambda_1 e^{-(\lambda_1-\mu_1)t}}{\lambda_1 - \mu_1 e^{-(\lambda_1-\mu_1)t}}\Big)^n.$$

Consequently,

$$P(Q > n) = E\Big[\frac{\lambda_1 - \mu_1}{\lambda_1 - \mu_1 e^{-(\lambda_1-\mu_1)T}} \Big(\frac{\lambda_1 - \lambda_1 e^{-(\lambda_1-\mu_1)T}}{\lambda_1 - \mu_1 e^{-(\lambda_1-\mu_1)T}}\Big)^n\Big] = E[g(T)], \qquad (12)$$

where the expectation is taken with respect to the exponential distribution of T. We shall look at the asymptotics of the above probability as n tends to infinity.

Fix $\epsilon > 0$ and define $t_1 = (1-\epsilon)\frac{\log n}{\lambda_1-\mu_1}$ and $t_2 = (1+\epsilon)\frac{\log n}{\lambda_1-\mu_1}$. We now decompose $E[g(T)]$ as $E[g(T)1(T < t_1)] + E[g(T)1(t_1 \le T \le t_2)] + E[g(T)1(T > t_2)]$. Now, it is easy to see that the function $g(t)$ is bounded above by 1 for all t, and so,

$$E[g(T)1(T > t_2)] \le P(T > t_2) = \exp\Big(-(1+\epsilon)\frac{\lambda-\mu}{\lambda_1-\mu_1}\log n\Big).$$

Recalling that $(\lambda-\mu)/(\lambda_1-\mu_1) = 1/\phi$, this implies that

$$n^{1/\phi}E[g(T)1(T > t_2)] \le n^{-\epsilon/\phi}, \tag{13}$$

which tends to zero as n tends to infinity.

It can be verified by differentiating $\log g(t)$ that it is an increasing function of t for all $n > \frac{\mu_1}{\lambda_1-\mu_1}$. Since we are considering large n asymptotics, we may thus assume that $\log g$, and hence g, are increasing functions. Therefore,

$$\begin{aligned} E[g(T)1(T < t_1)] \le g(t_1) &= \frac{\lambda_1-\mu_1}{\lambda_1-\mu_1 n^{-(1-\epsilon)}}\Big(\frac{\lambda_1-\lambda_1 n^{-(1-\epsilon)}}{\lambda_1-\mu_1 n^{-(1-\epsilon)}}\Big)^n \\ &\le \Big(1-\frac{(\lambda_1-\mu_1)n^{-(1-\epsilon)}}{\lambda_1-\mu_1 n^{-(1-\epsilon)}}\Big)^n \le \Big(1-\frac{(\lambda_1-\mu_1)}{\lambda_1}\frac{n^\epsilon}{n}\Big)^n. \end{aligned}$$

Hence,

$$n^{1/\phi}E[g(T)1(T < t_1)] \le n^{1/\phi}\exp\Big(-\frac{\lambda_1-\mu_1}{\lambda_1}n^\epsilon\Big), \tag{14}$$

which tends to zero as n tends to infinity.

We have thus shown that $n^{1/\phi}E[g(T)1(T < t_1)]$ and $n^{1/\phi}E[g(T)1(T > t_2)]$ both tend to zero as n tends to infinity. It only remains to consider $T \in [t_1, t_2]$. We have

$$E[g(T)1(t_1 \le T \le t_2) = \int_{t_1}^{t_2}(\lambda-\mu)e^{-(\lambda-\mu)t}g(t)dt,$$

where $g(t)$ is implicitly defined in (12). Making the change of variables $v = (\lambda_1-\mu_1)t - \log n$, we can rewrite the above integral as

$$\begin{aligned} E[g(T)1(t_1 \le T \le t_2)] &= \int_{-\epsilon\log n}^{\epsilon\log n}\frac{(\lambda-\mu)\exp(-\frac{v+\log n}{\phi})}{\lambda_1-\mu_1 e^{-(v+\log n)}}\Big(\frac{\lambda_1-\lambda_1 e^{-(v+\log n)}}{\lambda_1-\mu_1 e^{-(v+\log n)}}\Big)^n dv \\ &= (\lambda-\mu)n^{-1/\phi}\int_{-\epsilon\log n}^{\epsilon\log n}\frac{e^{-v/\phi}}{\lambda_1-\frac{\mu_1}{n}e^{-v}}\Big(\frac{\lambda_1-\frac{\lambda_1}{n}e^{-v}}{\lambda_1-\frac{\mu_1}{n}e^{-v}}\Big)^n dv. \end{aligned}$$

Rearranging, and taking limits as n tends to infinity, we get

$$\lim_{n\to\infty} n^{1/\phi}E[g(T)1(t_1 \le T \le t_2)] = \frac{\lambda-\mu}{\lambda_1}\int_{-\infty}^{\infty}e^{-v/\phi}\exp\Big(-\frac{\lambda_1-\mu_1}{\lambda_1}e^{-v}\Big)dv.$$

Now, change variables again, setting $x = \frac{\lambda_1-\mu_1}{\lambda_1}e^{-v}$. We get

$$\lim_{n\to\infty} n^{1/\phi}E[g(T)1(t_1 \le T \le t_2)] = \frac{\lambda-\mu}{\lambda_1-\mu_1}\int_0^\infty\Big(\frac{\lambda_1 x}{\lambda_1-\mu_1}\Big)^{\frac{1}{\phi}-1}e^{-x}dx. \tag{15}$$

Recall that the Gamma function is defined as $\Gamma(\alpha) = \int_0^\infty x^{\alpha-1}e^{-x}dx$ for $\alpha > 0$. We also saw in (14) and (13) that $n^{1/\phi}E[g(T)1(T < t_1)]$ and $n^{1/\phi}E[g(T)1(T > t_2)]$ tend to zero as n tends to infinity. Hence, we have from (12) and (15) that

$$\lim_{n\to\infty} n^{1/\phi}P(Q > n) = \frac{1}{\phi}\Big(\frac{\lambda_1}{\lambda_1-\mu_1}\Big)^{\frac{1}{\phi}-1}\Gamma\Big(\frac{1}{\phi}\Big) = \Big(\frac{\lambda_1}{\lambda_1-\mu_1}\Big)^{\frac{1}{\phi}-1}\Gamma\Big(\frac{1}{\phi}+1\Big).$$

This completes the proof of the theorem.

References

[1] David, H. Probability distribution of drug-resistant mutants in unselected populations of Mycobacterium tuberculosis. *Applied and Environmental Microbiology* **20**, 810 (1970).

[2] Shimao, T. Drug resistance in tuberculosis control. *Tubercle* **68**, 5 (1987).

[3] Luria, S. & Delbrück, M. Mutations of bacteria from virus sensitivity to virus resistance. *Genetics* **28**, 491 (1943).

[4] Lea, D. & Coulson, C. The distribution of the numbers of mutants in bacterial populations. *Journal of Genetics* **49**, 264–285 (1949).

[5] Zheng, Q. Progress of a half century in the study of the Luria–Delbrück distribution. *Mathematical biosciences* **162**, 1–32 (1999).

[6] Tan, W. On distribution theories for the number of mutants in cell populations. *SIAM Journal on Applied Mathematics* 719–730 (1982).

[7] Stewart, F., Gordon, D. & Levin, B. Fluctuation analysis: the probability distribution of the number of mutants under different conditions. *Genetics* **124**, 175 (1990).

[8] Ma, W., Sandri, G. & Sarkar, S. Analysis of the Luria-Delbrück distribution using discrete convolution powers. *Journal of Applied Probability* **29**, 255–267 (1992).

[9] Pakes, A. Remarks on the Luria-Delbrück distribution. *Journal of Applied Probability* **30**, 991–994 (1993).

[10] Angerer, W. An explicit representation of the Luria–Delbrück distribution. *Journal of Mathematical Biology* **42**, 145–174 (2001).

[11] Rosche, W. & Foster, P. Determining mutation rates in bacterial populations. *Methods* **20**, 4–17 (2000).

[12] Iwasa, Y., Nowak, M. & Michor, F. Evolution of resistance during clonal expansion. *Genetics* **172**, 2557 (2006).

[13] Komarova, N. Stochastic modeling of drug resistance in cancer. *Journal of theoretical biology* **239**, 351–366 (2006).

[14] Komarova, N., Wu, L. & Baldi, P. The fixed-size Luria–Delbruck model with a nonzero death rate. *Mathematical Biosciences* **210**, 253–290 (2007).

[15] Slutsky, E. Ueber stochastische asymptoten und grenzwerte. *Metron* **5**, 3–89 (1925).

[16] Nolan, J. P. *Stable Distributions - Models for Heavy Tailed Data* (Birkhäuser, Boston, 2010). In progress, Chapter 1 online at academic2.american.edu/$\sim$jpnolan.

[17] Lipsitch, M. & Levin, B. Population dynamics of tuberculosis treatment: mathematical models of the roles of non-compliance and bacterial heterogeneity in the evolution of drug resistance. *The International Journal of Tuberculosis and Lung Disease* **2**, 187–199 (1998).

[18] Reed, W. & Hughes, B. On the size distribution of live genera. *Journal of theoretical biology* **217**, 125–135 (2002).
